# Supplementary material for: Content-rich biological network constructed by mining PubMed abstracts
Source: BMC Bioinformatics. 2004 Oct 8;5:147. doi: 10.1186/1471-2105-5-147 (PMC528731; doi:10.1186/1471-2105-5-147)
Supplement: Additional File 2 — The original results of the above study (non-essential files are deleted to keep the file size under the limit set by BMC bioinformatics). [file 1471-2105-5-147-S2.bz2 › chilibotAdditionalFile2/dip40/698ID9287012E18025/html/FTSZ_FTSA.html]

 


 **FTSZ** and **FTSA** 
  
Found 107 abstracts in PubMed, retrieved 40.  
 

 What does Google say? 
 PDF only 
| .edu only 

---

**Interactive relationship** (e.g. stimulation, inhibition, etc)

**Neutral relationship**- We demonstrate for the first time a specific association between S. aureus  **FtsA**  and  **FtsZ**  proteins and self interaction of S. aureus  **FtsZ** .  Ref: 10753635 Biochem Biophys Res Commun, 2000
- We have validated and characterized this interaction by co expressing  **FtsA**  and  **FtsZ** , from both E. coli and S. aureus, in the yeast two hybrid system.  Ref: 10753635 Biochem Biophys Res Commun, 2000
- Further dissection of this motif by alanine scanning mutagenesis showed that Phe376 likely plays a major role in the  **FtsA**   **FtsZ**  interaction.  Ref: 10753635 Biochem Biophys Res Commun, 2000
- This work has important implications for the design of antagonists and agonists of the  **FtsA**   **FtsZ**  interaction as such agents could provide a novel approach for tackling multi resistant Gram positive pathogens.  Ref: 10753635 Biochem Biophys Res Commun, 2000
- **FtsA**  directly interacts with  **FtsZ**  and probably targets other proteins to the septum.  Ref: 11032797 EMBO J, 2000
- Characterization of  **FtsZ**  residues essential for the interactions with ZipA and with  **FtsA** .  Ref: 11278571 J Biol Chem, 2001
- A conserved residue at the extreme C terminus of  **FtsZ**  is critical for the  **FtsA**   **FtsZ**  interaction in Staphylococcus aureus.  Ref: 10753635 Biochem Biophys Res Commun, 2000
- Specific regions were identified which abrogated  **FtsZ**   **FtsZ**  binding but unaffected the  **FtsA**   **FtsZ**  interaction.  Ref: 11394911 Biochem Biophys Res Commun, 2001
- As in other organisms  **FtsA**  is probably recruited immediately after  **FtsZ** .  Ref: 11298280 Mol Microbiol, 2001
- These results suggest that the molecular mechanism of the  **FtsA** \* bypass is to stabilize  **FtsZ**  assembly via a parallel pathway.  Ref: 12634424 Proc Natl Acad Sci U S A, 2003
- Using two different in vivo assays, we found that the 12 amino acid truncation of  **FtsZ**  was defective in binding to  **FtsA** .  Ref: 10601211 J Bacteriol, 1999
- These mutant  **FtsZ**  proteins were analyzed for their interaction with both wildtype  **FtsZ**  and  **FtsA**  using the yeast two hybrid assay.  Ref: 11394911 Biochem Biophys Res Commun, 2001
- The septal ring in Escherichia coli consists of at least nine essential gene products whose order of assembly resembles a mostly linear dependency pathway  **FtsA**  and ZipA directly bind  **FtsZ**  polymers at the prospective division site, followed by the sequential addition of FtsK, FtsQ, FtsL, FtsW, FtsI, and FtsN.  Ref: 11948172 J Bacteriol, 2002
- Using deletion mutagenesis, we have shown that a highly conserved motif as small as 10 residues in the extreme C terminus of S. aureus  **FtsZ**  is critical for the interaction with  **FtsA** .  Ref: 10753635 Biochem Biophys Res Commun, 2000
- This suggests that ATP hydrolysis by  **FtsA**  is required, together with GTP hydrolysis by  **FtsZ** , for cell division in B. subtilis and possibly in most eubacteria.  Ref: 11298280 Mol Microbiol, 2001
- Two peptide protein interactions are known in this process  **FtsA**  and ZipA bind the C terminus of  **FtsZ** , the bacterial tubulin homologue, which is the first septal component to appear at the septum.  Ref: 11254984 Biochimie, 2001
- In Escherichia coli,  **FtsZ**  is required for the recruitment of the essential cell division proteins  **FtsA**  and ZipA to the septal ring.  Ref: 10601211 J Bacteriol, 1999
- **FtsA**  and ZipA localized into a division ring following the same pattern and time course as  **FtsZ** .  Ref: 12754232 J Bacteriol, 2003
- We have shown that a fusion, which encodes the last 70 residues of  **FtsZ**  in the two hybrid system, is sufficient for the interaction with  **FtsA**  and ZipA.  Ref: 11278571 J Biol Chem, 2001
- ZipA and  **FtsA**  are essential division proteins in Escherichia coli that are recruited to the division site by interaction with  **FtsZ** .  Ref: 11847116 EMBO J, 2002
- In addition, these strains have the ddlB, ftsQ,  **ftsA**  and  **ftsZ**  genes under the control of the IPTG inducible promoter P tac, allowing to control artificially  **ftsZ**  expression for normal cell division to take place.  Ref: 11254983 Biochimie, 2001
- **FtsA**  is present in much higher quantities in B. subtilis than in Escherichia coli, with approximately one molecule of  **FtsA**  for five of  **FtsZ** .  Ref: 11298280 Mol Microbiol, 2001
- These results indicate that MinCD blocks the assembly of the septation machinery at a later step than SulA, at the stage at which  **FtsA**  is added to the  **FtsZ**  ring.  Ref: 10931335 Mol Microbiol, 2000
- Filamentation was correlated with reduced  **FtsZ**  levels and could be specifically suppressed by overexpression of ftsQI,  **ftsA**  and  **ftsZ** , and to some extent by  **ftsZ**  alone.  Ref: 10572302 Biochimie< MedlineTA>Biochimie, 2000

**Non-interactive relationship** (e.g. studied together, co-existance, homology, etc.)

- The promoter, ftsA1p, located in the ftsQ coding sequence, co regulates  **ftsA**  and  **ftsZ** .  Ref: 10564486 Mol Microbiol, 1999
- The organization of the two chromosomal regions in P. aeruginosa and E. coli is remarkably similar with the following gene order pbp3 pbpB, murE, murF, mraY, murD, ftsW, murG, murC, ddlB, ftsQ,  **ftsA** ,  **ftsZ** , and envA LpxC.  Ref: 11281713 Protein Expr Purif, 2001
- Concentration and Assembly of the Division Ring Proteins  **FtsZ** ,  **FtsA** , and ZipA during the Escherichia coli Cell Cycle.  Ref: 12754232 J Bacteriol, 2003
- Mutations surrounding Asp 373 differentially affected the interactions of  **FtsZ**  with ZipA and  **FtsA** , indicating that these proteins bind the C terminus of  **FtsZ**  differently.  Ref: 11278571 J Biol Chem, 2001
- GFP FtsQ localization to the septum depended on the cell division proteins  **FtsZ**  and  **FtsA** , which are cytoplasmic, but not on FtsL and FtsI, which are bitopic membrane proteins with comparatively large periplasmic domains.  Ref: 9882666 J Bacteriol, 1999
- By the coexpression of the E. coli  **ftsA**  and  **ftsZ**  genes, which encode key proteins in cell division, growth of recombinant strains as well as production of human leptin and human insulin like growth factor I was improved.  Ref: 12571061 Appl Environ Microbiol, 2003
- Zonal synthesis can be triggered in  **ftsA** , ftsQ and ftsI pbpB division mutants growing as filaments at the restrictive temperature, but not in  **ftsZ**  mutant strains.  Ref: 11782501 Microbiology, 2002
- Compared with the parent strain and in contrast to ftsQ,  **ftsA**  and  **ftsZ**  mutants, pbpB Ts cells growing at the permissive temperature 28 degrees C showed a long D period 42 min versus 18 min in the parent indicative of an extended segregation time.  Ref: 10476030 Mol Microbiol, 1999
- Septal localization depended upon every other division protein tested  **FtsZ** ,  **FtsA** , FtsQ, and FtsL.  Ref: 9882665 J Bacteriol, 1999
- The concentration of the cell division proteins  **FtsZ** ,  **FtsA** , and ZipA and their assembly into a division ring during the Escherichia coli B r K cell cycle have been measured in synchronous cultures obtained by the membrane elution technique.  Ref: 12754232 J Bacteriol, 2003
- The gonococcal dcw cluster consists of 17 genes, in the order 5 mraZ mraW ftsI murE hyp1 murF mraY hyp2 murD ftsW murG murC ddl ft sQ  **ftsA**   **ftsZ**  hyp3 3.  Ref: 10876091 Gene, 2000
- Regulation of Escherichia coli cell division genes  **ftsA**  and  **ftsZ**  by the two component system rcsC rcsB.  Ref: 10564486 Mol Microbiol, 1999
- These localization results support the model that E. coli cell division proteins assemble sequentially as a multimeric complex at the division site first  **FtsZ** , then  **FtsA**  and ZipA independently of each other, followed successively by FtsK, FtsQ, FtsL, FtsW, FtsI and FtsN.  Ref: 11703663 Mol Microbiol, 2001
- FtsL localization is dependent upon the function of  **FtsZ** ,  **FtsA**  and FtsQ, but not FtsI.  Ref: 10027987 Mol Microbiol, 1999
- ZipA and  **FtsA**  are recruited independently to the  **FtsZ**  cytokinetic ring Z ring and are essential for cell division of Escherichia coli.  Ref: 12634424 Proc Natl Acad Sci U S A, 2003
- ZipA requires  **FtsZ** , but not  **FtsA**  or FtsI, to be localized, indicating that it is recruited by  **FtsZ** .  Ref: 10209756 Mol Microbiol, 1999
- Division can be restored in the absence of PBP2 activity by increasing the concentration of the cell division proteins FtsQ,  **FtsA** , and  **FtsZ** .  Ref: 11102351 Genetics, 2000
- These regions of actin and tubulin, which are not present in their prokaryotic counterparts  **FtsA**  and  **FtsZ** , are involved in the polymerization of the two cytoskeletal proteins.  Ref: 11580270 J Struct Biol, 2001
- In Escherichia coli, at least nine essential gene products participate directly in septum formation  **FtsA** , FtsI, FtsL, FtsK, FtsN, FtsQ, FtsW,  **FtsZ**  and ZipA.  Ref: 11703663 Mol Microbiol, 2001
- Because another proposed function of  **FtsZ**  is the recruitment of  **FtsA**  and ZipA, we tested whether the C terminal core domain was important for interactions with these proteins.  Ref: 10601211 J Bacteriol, 1999
- Transcriptional start sites upstream of ftsQ,  **ftsA**  and  **ftsZ**  were determined by primer extension and six promoters were identified.  Ref: 10876091 Gene, 2000
- It is well documented that  **FtsA**   **FtsZ**  co localization at the septum of dividing bacteria is essential for successful cell division in E. coli.  Ref: 10753635 Biochem Biophys Res Commun, 2000
- Observation of cell morphology revealed that the coexpression of the  **ftsA**  and  **ftsZ**  genes successfully suppressed filamentation caused by the accumulation of recombinant proteins.  Ref: 12571061 Appl Environ Microbiol, 2003
- A region of the dcw division cell wall cluster, including ftsQ,  **ftsA** ,  **ftsZ**  and murC, was sequenced in DX and SIN strains as a basis for studying cell division.  Ref: 12429070 BMC Microbiol, 2002
- The results imply that association of both  **FtsA**  and ZipA with  **FtsZ**  polymers is needed for further maturation of the nascent organelle.  Ref: 11948172 J Bacteriol, 2002
- Examination of cells expressing different combinations of the fusions indicated that  **FtsA** , FtsQ, FtsL and FtsI co localize with  **FtsZ**  in filaments depleted of FtsN.  Ref: 11703663 Mol Microbiol, 2001
- three promoters were located upstream of  **ftsZ**  in the intergenic space, two were upstream of  **ftsA**  within ftsQ and one was upstream of ftsQ within ddl.  Ref: 10876091 Gene, 2000
- We also constructed translational fusions of  **FtsZ** ,  **FtsA** , FtsQ, FtsL and FtsI to enhanced cyan or yellow fluorescent protein ECFP or EYFP respectively, GFP variants with different fluorescence spectra.  Ref: 11703663 Mol Microbiol, 2001
- Expression of ftsA1p, the  **ftsA**   **ftsZ**  promoter submitted to RcsB stimulation, is greatly increased in mucM mutants, and this expression is dependent on RcsB and ftsA1p.  Ref: 11425476 FEMS Microbiol Lett, 2001
- A chromosomal 10355 bp segment of Enterococcus hirae S185 contains nine orfs which occur in the same order as the MraW, FtsL, PBP3, MraY, MurD, MurG, FtsQ,  **FtsA**  and  **FtsZ**  encoding genes of the division and cell wall clusters of Escherichia coli and Bacillus subtilis.  Ref: 10520745 DNA Seq, 1998
- Selected amplification of the cell division genes ftsQ  **ftsA**   **ftsZ**  in Escherichia coli.  Ref: 11102351 Genetics, 2000
- Gene  **ftsA**  is not adjacent to  **ftsZ**  or other genes of the cloned fragment.  Ref: 10679194 Biochem Biophys Res Commun, 2000
- Mucoidy results from the activation of cps genes and mecillinam resistance from  **ftsA**   **ftsZ**  overexpression, both traits caused by the increased activity of the RcsB effector.  Ref: 11425476 FEMS Microbiol Lett, 2001
- This result indicates that the reduction in  **ftsZ**  transcription observed when the promoters in the ddlB  **ftsA**  region are disconnected from the upstream sequences of the dcw cluster as observed by Flärdh et al.  Ref: 11254983 Biochimie, 2001
- Among partners of  **FtsZ**  the  **FtsA**  protein and a globular part of the SpoIIE protein were identified.  Ref: 11830939 Folia Microbiol Praha , 2001
- These observations are consistent with the conserved role of  **FtsA**  and  **FtsZ**  in bacterial cell division.  Ref: 10753635 Biochem Biophys Res Commun, 2000
- It comprises 15 open reading frames, of which the organization is mraZ mraW ftsL ftsI murE murF mraY murD ftsW murG murC ftsQ  **ftsA**   **ftsZ**  envA, in that order.  Ref: 12153713 J Biochem Tokyo , 2002
- Since these results remain unexplained, we examined an  **ftsZ**  mutant and other cell division mutants  **ftsA** , ftsQ, and ftsI and a PBP2 mutant for induction of beta lactamase.  Ref: 12107141 J Bacteriol, 2002
- septal localization of FtsW required  **FtsZ** ,  **FtsA** , FtsQ, and FtsL but not FtsI.  Ref: 11807049 J Bacteriol, 2002
- Specifically, cephalexin acylated PBP3 about 50% faster in a population of dividing cells than in a population of filamentous cells in which division was inhibited by inactivation or depletion of  **FtsZ** ,  **FtsA** , FtsQ, FtsW, or FtsN.  Ref: 12813065 J Bacteriol, 2003
- **FtsZ** , a tubulin like protein with GTPase activity, and  **FtsA** , an actin like protein with ATPase activity, are two proteins known to play critical roles in the later stages of the bacterial cell cycle.  Ref: 10753635 Biochem Biophys Res Commun, 2000
- Consistent with a direct interaction between  **FtsZ**  and the MinCD inhibitor, we find that increased  **FtsZ** , but not  **FtsA** , suppresses MinCD induced lethality.  Ref: 11673433 J Bacteriol, 2001
- In this paper, size exclusion chromatography was used to study the effect of the SulA and MinCD division inhibitors on the oligomerization state of endogenous  **FtsZ**  in cytoplasmic extracts, and immunofluorescence microscopy was used to determine the effect of SulA and MinCD on the formation of  **FtsZ** ,  **FtsA**  and ZipA rings at potential division sites.  Ref: 10931335 Mol Microbiol, 2000
- In a reverse approach, we use fusions of green fluorescent protein GFP to  **FtsZ** ,  **FtsA**  and ZipA to show that these proteins localize to the division site in an FtsL independent fashion.  Ref: 10027987 Mol Microbiol, 1999
- Consistent with this, we found that an  **FtsZ**  mutant unable to interact with both ZipA and  **FtsA**  was unable to assemble into Z rings.  Ref: 11847116 EMBO J, 2002
- We tried to identify regulators of the ftsQ  **ftsA**   **ftsZ**  operon among mecillinam resistant mutants, which include strains overexpressing these genes.  Ref: 11102351 Genetics, 2000
